# Dementia Care-Related Stress and Working Memory Performance: Examining the Mediating Role of Sleep

**DOI:** 10.1093/geroni/igab046.1739

**Published:** 2021-12-17

**Authors:** Ashley Blasi, Francesca Falzarano

**Affiliations:** 1 Fordham University, Bronx, New York, United States; 2 Weill Cornell Medicine, Douglaston, New York, United States

## Abstract

Dementia Family caregivers often experience significant stress and burden, which has been associated with a myriad of adverse effects on physical and mental health, as well as cognition. The impact of caregiving on health and well-being may have negative implications on the provision of quality and effective care. Specifically, working memory is a key domain of cognition that ultimately underlies logic and decision making processes. Thus, the purpose of the current study is to examine the associations between dementia care-related stress and working memory, as well as potential mediators of this relationship, in a sample of 50 primary caregivers who completed measures examining stress, including burden and overload, and several domains of cognition. Our results showed that higher levels of caregiving overload were associated with worse working memory performance, measured using the N-back task. Additionally, we found that lower sleep quality fully mediated the relationship between overload and working memory performance, such that the negative effects of overload on working memory performance may operate as a result of sleep impairment. By determining the mediating role of sleep while also providing evidence to support the negative relationship between stress and working memory, our results provide support for the development of interventions that target factors such as burden and sleep quality to help mitigate stress in caregivers.

